# Prevalent and new use of common drugs for the incidence of community-acquired acute kidney injury: cohort and case-crossover study

**DOI:** 10.1038/s41598-024-66532-w

**Published:** 2024-08-02

**Authors:** Miho Kimachi, Tatsuyoshi Ikenoue, Shingo Fukuma

**Affiliations:** 1 Human Health Sciences, Kyoto University Graduate School of Medicine, 53 Shogoin-Kawara-cho, Sakyo-ku, Kyoto, 606-8507 Japan; 2 Shiga University Center for Data Science Education and Research, Shiga, Japan; 3 Department of Epidemiology Infectious Disease Control and Prevention, Hiroshima University Graduate School of Biomedical and Health Sciences, Hiroshima, Japan

**Keywords:** Public health, Nephrology

## Abstract

Although community-acquired acute kidney injury (CA-AKI) represents a significant subset of all AKI incidence, evidence is limited due to the lack of comprehensive data prior to diagnosis. Here, we examined the risk of drug use for CA-AKI by using exhaustive pre-diagnostic prescription data. We included 78,754 working-age healthy individuals who underwent an annual health checkup program. We conducted a cohort study to assess the association between prevalent drug use and subsequent CA-AKI incidence using the Cox proportional hazard model. Subsequently, we conducted a case-crossover study to compare the new drug use in the case period directly before the CA-AKI incidence (− 3 to 0 months) with that in the control period far before the CA-AKI incidence (− 15 to − 12 months and − 9 to − 6 months) using the conditional Poisson regression model. The prevalent use of renin–angiotensin–aldosterone system (RAAS) inhibitors was associated with an increased CA-AKI incidence, but the new use was not. The new use of diuretics, anti-infectious drugs, and contrast medium was also associated with an increased CA-AKI incidence. These results suggest we need to pay attention for the incidence of AKI among the general population taking those common drugs.

## Introduction

The incidence of community-acquired acute kidney injury (CA-AKI) among the general population, as well as that of hospital-acquired AKI (HA-AKI), has been increasing globally^1,2^. Given the increasing number of recent studies on CA-AKI and HA-AKI, most studies suggest that CA-AKI should be distinguished from HA-AKI because they have different baseline conditions, risk factors, and clinical courses; however, the evidence regarding CA-AKI is currently insufficient due to the lack of comprehensive data prior to diagnosis. Furthermore, CA-AKI has been reported to be more preventable than HA-AKI.^3–5^ Since substantial AKI is irreversible and advances to chronic kidney disease (CKD) due to the progression of tubular damage and interstitial fibrosis^6–10^, it is essential to identify risk factors of CA-AKI to prevent it.

CA-AKI is often caused by prerenal failure due to decreased renal perfusion accompanied by vomiting, diarrhoea, diaphoresis, and bleeding; it is also caused by intrinsic nephric failure due to tubular or interstitial injuries accompanied by the nephrotoxic effects of several drugs, contrast media, and other invasive events, including sepsis^1,11^. Especially, several drugs prescribed in primary care settings, such as antihypertensive drugs, including renin–angiotensin–aldosterone system (RAAS) inhibitors, gastrointestinal drugs, including proton pump inhibitors (PPIs), and non-steroidal anti-inflammatory drugs (NSAIDs), are commonly prescribed in primary care settings and are likely to be associated with the incidence of CA-AKI^12–20^. However, most studies have examined the association between these drugs and CA-AKI incidence using patient data obtained from a hospital that may overestimate risks, and evidence in the general population has not been sufficiently established. Additionally, most studies assessed only the short-term effects of risk factors on the incidence of CA-AKI. It is essential to modify the precaution for each treatment depending on the timing that could increase CA-AKI risk.

This study aimed to assess the association between potential risk factors and the incidence of CA-AKI in the general population. Using a cohort design and case-crossover design, we evaluated whether the prevalent and new drug use prescribed in primary care settings are risk factors for CA-AKI.

## Results

### Study subjects and baseline characteristics

**Figure 1:**
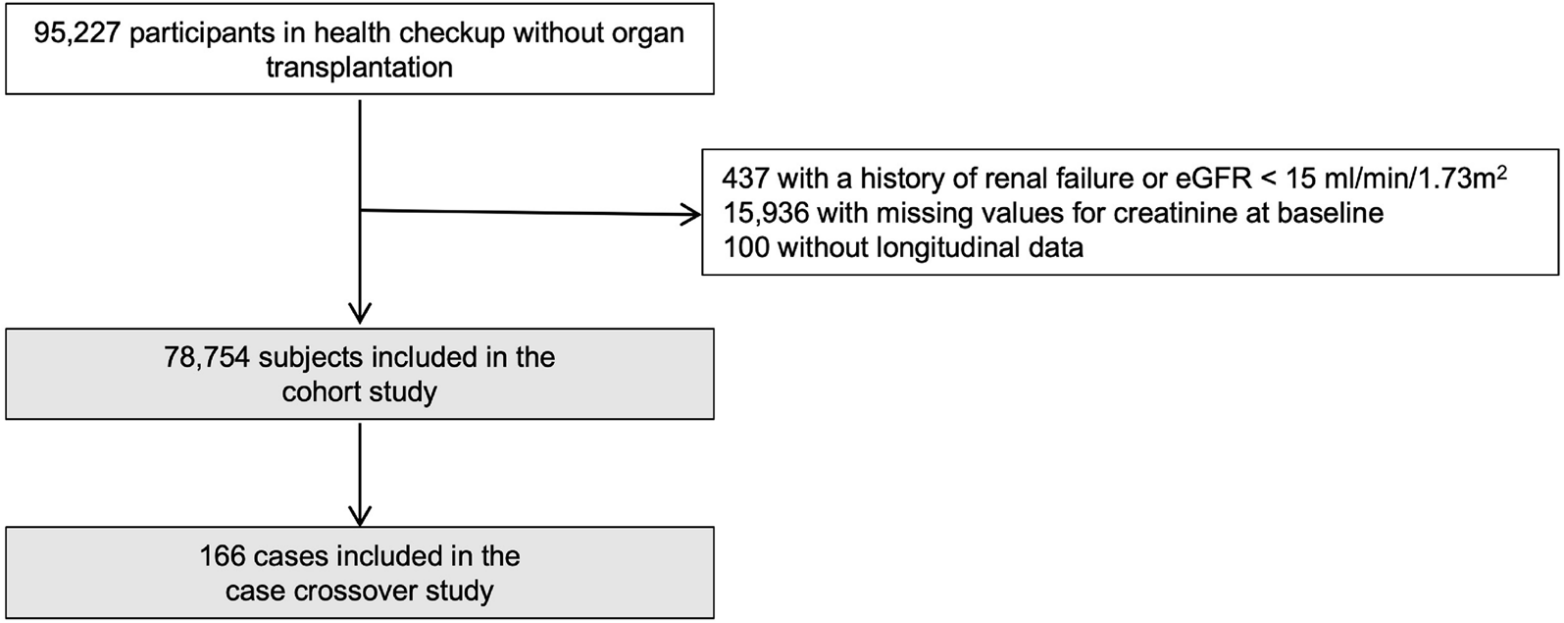
Study flow diagram and study selection process.

A total of 95,227 individuals participated in the health checkup. Of these, 16,473 were excluded—437 had a history of renal failure or an estimated glomerular filtration rate (eGFR) < 15 mL/min/1.73 m^2^, 15,936 individuals did not have data of creatinine at baseline, and 100 did not have longitudinal data. Ultimately, 78,754 individuals were analysed in the cohort design (Fig. 1). A total of 166 patients (0.21%) developed CA-AKI during the follow-up period {5.36 (95% confidence intervals [95% CIs], 4.60–6.24 per 10,000 person-years)}.

As shown in Table 1, the mean age was 53.0 (standard deviation [SD], 10.5) years, 26.2% were female, and the mean baseline eGFR value was 77.4 (SD, 15.2) mL/min/1.73 m^2^. Subjects with subsequent CA-AKI tended to be male, have more frequent smoking and less regular exercise habits, have a higher frequency of chronic liver disease (CLD), and be prescribed RAAS inhibitors and antidiabetic agents. The baseline renal function measured by eGFR did not differ markedly between groups. We did not describe values of albumin and C-reactive protein (CRP) because of missing data in more than 60% of subjects.

**Table 1:** Baseline characteristics of subjects according to the presence or absence of CA-AKI incidence.

Characteristics	Total (n = 78,754)	CA-AKI (+) (n = 166)	CA-AKI (−) (n = 78,588)	*p*-value
Age (years), mean (SD)	53.0 (10.5)	53.1 (10.0)	53.0 (10.5)	0.88
Female, %	26.2	11.5	26.2	0.001 >
Body mass index, mean (SD)	24.6 (4.0)	25.1 (5.0)	24.6 (4.0)	0.17
Smoking, %	27.8	43.6	27.8	0.001 >
Alcohol (everyday), %	36.1	40.1	36.1	0.29
Regular exercise, %	24.3	13.9	24.3	0.002
Systolic blood pressure, mmHg mean (SD)	129.7 (18.2)	132.3 (19.2)	129.7 (18.2)	0.063
Diastolic blood pressure, mmHg mean (SD)	80.1 (13.0)	81.7 (13.2)	80.1 (13.0)	0.12
Comorbidities, %				
Stroke	3.7	2.4	3.7	0.37
Ischaemic heart disease	9.0	9.6	9.0	0.78
Chronic heart failure	6.6	7.8	6.6	0.51
Chronic liver disease	20.0	26.5	20.0	0.035
Laboratory data, mean (SD)				
eGFR, ml/min/1.73 m^2^	77.4 (15.2)	76.8 (17.6)	77.4 (15.2)	0.59
Creatinine, mg/dL	0.80 (0.18)	0.85 (0.25)	0.80 (0.18)	0.001 >
Haemoglobin, g/dL	14.6 (1.5)	14.5 (1.5)	14.6 (1.5)	0.47
Total protein, g/dL	7.3 (0.42)	7.3 (0.51)	7.3 (0.42)	0.42
ALT, U/L	28.2 (23.0)	29.6 (22.5)	28.2 (23.0)	0.43
AST, U/L	25.8 (38.5)	29.6 (18.1)	25.8 (38.5)	0.20
Γ-GTP,	53.7 (79.7)	83.9 (157.7)	53.6 (79.4)	0.001 >
Uric acid, mg/dL	5.8 (1.5)	6.0 (1.5)	5.8 (1.5)	0.050
LDL cholesterol, mg/dL	123.9 (32.1)	115.6 (38.0)	123.9 (32.1)	0.001 >
HDL cholesterol, mg/dL	60.5 (16.9)	57.6 (17.9)	60.5 (16.9)	0.025
Triglyceride, mg/dL	132.6 (129.1)	169.3 (169.2)	132.5 (129.0)	0.001 >
HbA1c, %	5.8 (0.88)	6.0 (1.1)	5.8 (0.88)	0.032
HbA1c over 6.5%	13.4	19.8	13.4	0.017
Urine protein				0.001 >
3 +	0.39	3.0	0.39	
2 +	1.4	1.8	1.4	
1 +	4.0	7.2	4.0	
Trace	9.1	10.2	9.1	
Negative	85.1	77.7	85.1	
Prescription, %				
RAAS inhibitor	26.4	35.5	26.4	0.007
Antihypertensive agent other than RAAS inhibitor	30.8	33.1	30.8	0.51
Diuretic	3.7	4.2	3.7	0.73
Antidiabetic agent	7.9	13.3	7.9	0.011
Proton pump inhibitor	19.2	19.3	19.2	0.98
Histamine-2 receptor antagonist	13.8	13.3	13.8	0.83
Active vitamin D3 analogue	1.2	1.8	1.2	0.48

Normally distributed continuous data were summarised as the mean (standard deviation), continuous variables with skewed data were summarised as median (interquartile range) and dichotomous or categorical data were summarised as proportions.

CA-AKI, community-acquired acute kidney injury; eGFR, estimated glomerular filtration rate; ALT, alanine aminotransferase; AST, aspartate aminotransferase; γ-GTP, γ-glutamyl transpeptidase; HbA1c, haemoglobin A1c; and RAAS, renin–angiotensin–aldosterone system.

A total of 15,936 subjects were excluded because of the unavailability of data regarding baseline creatinine; these subjects were likely to be older, less commonly males, have more frequent smoking and regular exercise habits, and have more prescriptions of RAAS inhibitors and other antihypertensive agents (see Supplementary Table S1).

### Association between the prevalent drug use and CA-AKI incidence

The maximum length of follow-up was 6.58 years, and the median length was 4.37 (interquartile range [IQR], 2.10–5.83) years. As shown in Table 2, the prevalent use of RAAS inhibitors was associated with CA-AKI incidence. Additionally, CA-AKI incidence was significantly associated with risk factors, including male sex, lower BMI, smoking, lower frequency of regular exercise, lower haemoglobin levels, and proteinuria. For sensitivity analyses, adjusting for fewer numbers of covariates, we confirmed the robustness of these findings, which were consistent with that of the main analysis (see Supplementary Table S2). Furthermore, we confirmed the continuation rates of each drug during the follow-up (Table 3). We performed sensitivity analyses, excluding histamine-2 receptor antagonists (H2Ras) with a continuation rate < 50% one year from the beginning of the study, excluding drugs other than RAAS inhibitors and other antihypertensive agents from covariates at the median time (4.37 years) of follow-up. We also confirmed the robustness of these findings, which were consistent with that of the main analysis (see supplementary Table S3).

**Table 2:** Association between CA-AKI incidence and baseline risk factors including the prevalent drug use (Model 1) (n = 78,753).

	Hazard ratio	95% Confidence interval	*p*-value
Continued use of common drugs			
RAAS inhibitor	1.59	(1.03–2.46)	0.037
Antihypertensive agent other than RAAS inhibitor	0.87	(0.57–1.33)	0.53
Diuretic	0.82	(0.37–1.80)	0.62
Antidiabetic agent	1.37	(0.80–2.34)	0.26
Proton pump inhibitor	1.03	(0.69–1.55)	0.88
Histamine-2 receptor antagonist	1.08	(0.68–1.70)	0.75
Active vitamin D3 analogue	2.95	(0.91–9.54)	0.071
Age (every 10 years old)	1.12	(0.94–1.34)	0.21
Female	0.26	(0.14–0.47)	0.001 >
eGFR			
≥ 90 mL/min/1.73 m^2^	Ref		
≥ 60 < 90 mL/min/1.73 m^2^	0.97	(0.65–1.46)	0.88
< 60 mL/min/1.73 m^2^	1.02	(0.55–1.89)	0.95
Proteinuria	1.70	(1.04–2.78)	0.033
Stroke	0.68	(0.25–1.85)	0.45
Ischaemic heart disease	1.01	(0.58–1.75)	0.99
Chronic heart failure	1.19	(0.65–2.19)	0.58
Chronic liver disease	1.39	(0.97–1.99)	0.07
Body mass index			
< 18.5	3.12	(1.61–6.07)	0.001
≥ 18.5 < 25	Ref		
≥ 25	1.05	(0.75–1.49)	0.76
Smoking	1.79	(1.29–2.49)	0.001
Alcohol (everyday)	0.94	(0.68–1.32)	0.73
Regular exercise	0.55	(0.35–0.87)	0.01
High blood pressure	1.07	(0.76–1.49)	0.76
Haemoglobin	0.75	(0.66–0.84)	0.001 >
Total protein	1.31	(0.89–1.94)	0.17
Urine acid	1.02	(0.90–1.14)	0.71
Dyslipidaemia	1.17	(0.85–1.62)	0.34
HbA1c ≥ 6.5%	1.16	(0.73–1.85)	0.54

RAAS, renin–angiotensin–aldosterone system; eGFR, estimated glomerular filtration rate; and HbA1c, haemoglobin A1c.

**Table 3:** Cumulative proportion of continuously prescribed medications, (%).
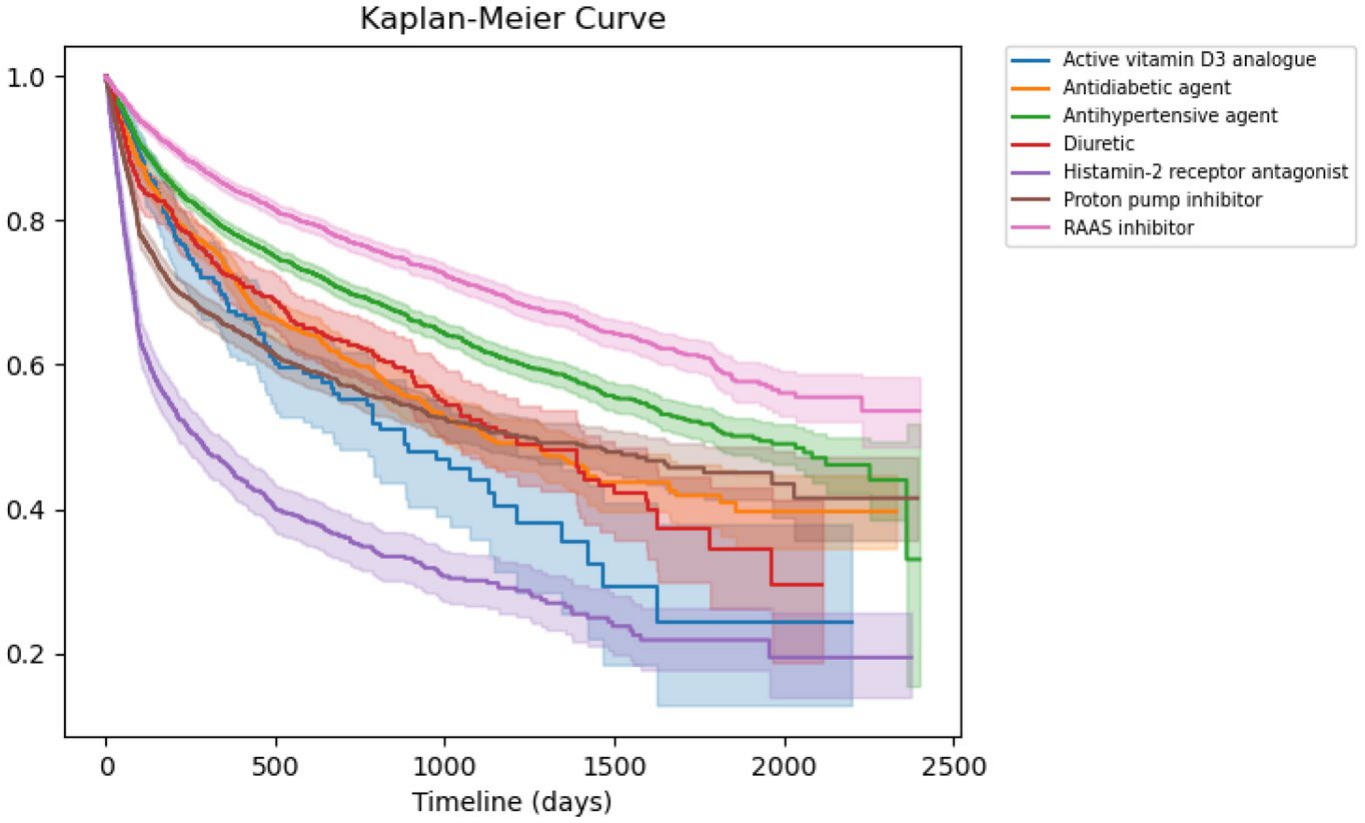

Common drugs	1 year	2 year	3 year	4 year	5 year	6 year	Median time
RAAS inhibitor	84.7	77.0	70.9	64.6	58.8	55.5	63.1
Antihypertensive agent other than RAAS inhibitor	78.6	69.7	62.4	56.2	50.4	46.1	54.5
Diuretic	72.4	62.8	52.3	43.2	34.5	29.6	41.1
Antidiabetic agent	73.0	60.4	51.2	43.7	40.9	39.7	43.7
Proton pump inhibitor	65.3	56.9	51.6	48.6	45.0	41.5	46.7
Histamine-2 receptor antagonist	45.7	35.4	30.1	24.9	21.8	19.4	21.8
Active vitamin D3 analogue	67.4	55.3	44.1	32.5	24.4	24.4	29.2

Kaplan–Meier Curves show the cumulative proportions of continued prescription by drug types. Discontinuation of a drug was defined as more than 90 days without a prescription since the last prescription period. The Table shows the percentage of drug continuation from the baseline at each time point from year 1 to year 6 and at the median time of follow-up.

RAAS, renin–angiotensin–aldosterone system.

### Association between the new drug use and CA-AKI incidence

We compared the incidence of CA-AKI between the case period (− 3 to 0 months) and control periods (− 15 to − 12 months and − 9 to − 6 months) of 166 subjects. As shown in Table 4, the subjects in which CA-AKI occurred received the new use of diuretics, anti-infectious drugs, and contrast medium during the case period compared with the control periods. Additionally, we compared the case period with only the earlier control period (− 15 to − 12 months) as a sensitivity analysis. This result was consistent with that of the main analysis (see Supplementary Table S4).

**Table 4:** Comparison of the new drug use between the case (− 3 to 0 months) and control periods (− 15 to − 12 months and − 9 to − 6 months), n = 166.

Common drugs	Incidence rate ratio	95% Confidence interval	*p*-value
RAAS inhibitor	1.11	(0.89–1.38)	0.37
Antihypertensive agent other than RAAS inhibitor	0.97	(0.76–1.24)	0.82
Diuretic	1.54	(1.20–1.98)	0.001
Antidiabetic agent	1.03	(0.81–1.32)	0.80
Proton pump inhibitor	1.23	(0.98–1.55)	0.071
Histamine-2 receptor antagonist	1.00	(0.75–1.35)	0.98
Active vitamin D3 analogue	0.85	(0.54–1.36)	0.51
Non-steroidal anti-inflammatory drug	1.22	(0.95–1.57)	0.12
Anti-infectious drug	1.78	(1.38–2.30)	0.001 >
Contrast medium	1.61	(1.27–2.03)	0.001 >

RAAS, renin–angiotensin–aldosterone system.

## Discussion

We included working-age individuals who underwent the national health checkup program in Japan. The prevalent use of RAAS inhibitors was associated with subsequent CA-AKI incidence, whereas the new use of RAAS inhibitors was not. Furthermore, the new use of diuretics, anti-infectious drugs, and contrast media was significantly associated with CA-AKI incidence. These results suggest that the potential risks for CA-AKI due to drug use and risk profiling may be useful for CA-AKI prevention.

This is the first study that exhaustively examined the association between drugs prescribed in primary care settings and CA-AKI incidence, considering two different timings (prevalent and new use) using different study designs (cohort and case-crossover designs). First, in the cohort design, the annual health checkup data allowed for the assessment of the long-term risks of CA-AKI. The present cohort study revealed an association between the prevalent use of RAAS inhibitors and CA-AKI incidence. RAAS inhibitors are widely prescribed as first-line antihypertensive agents because of the established evidence supporting their organ-protective effects, including the prevention of renal failure, proteinuria, and cardiovascular disease^21,22^. Generally, the incidences of AKI and hyperkalaemia have been reported as the side effects of the new use of RAAS inhibitors, especially for patients with CKD^23^. Our findings did not reveal the association between the new use of RAAS inhibitors and CA-AKI incidence because our study used health checkup data and probably included a well-tolerated cohort population. Additionally, it might cause reporting bias because the patients often indicate the initial dip of renal function when the RAAS inhibitors were newly prescribed. Nevertheless, our study revealed an association between the prevalent use of RAAS inhibitors and CA-AKI incidence. This finding suggests that physicians need to monitor patients regularly and continuously for dehydration during the prescription of RAAS inhibitors even for patients with normal kidney function because RAAS inhibitors sustainably reduce intraglomerular pressure, which can increase the risk of low eGFR and CA-AKI by several events accompanied by acute volume depletion^13,14,24^.

In contrast, our case-crossover study revealed the associations of the new use of diuretics, anti-infectious drugs, and contrast media with CA-AKI incidence. This study design allowed the self-matching of many covariates, although the number of outcomes was limited. Anti-infectious drugs and contrast media have been reported to cause acute tubulointerstitial nephritis and AKI, accompanied by allergic reactions, even with the use of low dosages for short periods. Additionally, the similar association have been reported for PPI and NSAIDs, although there were no significant differences in this study. This disorder could also occur in individuals without underlying diseases, such as renal dysfunction^15–20^. More careful monitoring is needed, especially immediately after the administration of these drugs, regardless of kidney function. Therefore, this study suggests that physicians need to modify the precautionary measures for each treatment depending on the timing of the increased risk of CA-AKI incidence.

CA-AKI incidence in the general population, that is, among those who underwent a health checkup program, was lower than the estimated rate of previous studies^1,3,25^. We should be careful about the overestimation of CA-AKI incidence from patient data because these patients are at high risk of AKI due to comorbidities and medical interventions^1,24^. Although it is usually difficult to examine pre-hospital risk factors in healthy populations without diagnosing CKD and other diseases, we had a unique opportunity to examine multiple risk factors, which were measured in the annual national health checkup system and medical claims data in Japan^26–28^. The present study, which used health checkup data, suggests that attention should be paid to subjects with risk profiling, such as more frequent smoking or less frequent exercise habit, in addition to male sex, lower BMI, proteinuria, and anaemia that were also recognized as the risk of HA-AKI. Further, we need to pay attention to the new use of diuretics, and anti-infectious drugs prescribed in primary care settings. Future studies should examine the association between CA-AKI and other comorbidities such as CHF, CLD, diabetes mellitus, hypertension, and pre-existing declined kidney function in more detail that are risk factors for HA-AKI, even though there was no significant association in this study^29–32^. Additionally, our study indicated the association between CA-AKI and the use of contrast medium as similar with HA-AKI. Previous study dose not necessarily reccomend the prior preparation for patients with normal kidney function. We should discuss more in the future study because of the conflicting evidence^33^.

This study has some limitations. First, several comorbidities and outcomes in the present study were defined based on the 10th revision of the International Classification of Diseases (ICD-10) codes, but not based on definitions from several recommendations or criteria, such as the Kidney Disease Improving Global Outcomes (KDIGO) clinical practice guidelines. Therefore, these data are likely to be under-detected, leading to misclassification^34,35^. Second, we were unable to assess the compliance of the participants for each drug. We described how medications were continuously prescribed (Table 3). Although some patients may restart their medication after the first discontinuation, we were unable to analyze changes in prescription patterns after the initial discontinuation. Third, we were unable to assess the influence of individual drugs, such as aminoglycoside antibiotics, included in the group of anti-infectious drugs because of the small number of events. Additionally, we were unable to determine whether events related to drugs had a greater impact on CA-AKI incidence. Drug prescription may be a surrogate marker of some events (see Supplementary Table S5). However, we were unable to adjust for these events because of the small number of events in the control group. Forth, we were unable to assess the influence of several relevant covariates, such as albumin and CRP levels, because of the high frequency of missing values. However, we considered that the missing values did not markedly influence our findings, because we confirmed that the results before and after multiple imputation, including albumin and CRP, were consistent (data not shown). Additionally, we could not extract the past history of cancer from the health check-up data. Moreover, we need to assess the influence of confounding by indication in future research using the analytical methods such as the instrumental variable method, because we could not perform the assessment in the present study because of loss of power. Fifth, we defined the date of diagnosis in the claims databases as the incident data of CA-AKI and other related events. If the diagnoses were delayed, the incident data was also delayed than actual periods. However, we considered that a reversal causality was unlikely to occur because of the sudden onset like CA-AKI. Finally, participants in the present study were limited to workers belonging to a specific occupation and their families, which might reduce generalizability.

We conclude that both the prevalent use and new use of several drugs that are commonly prescribed in the primary care setting might contribute to CA-AKI development. We need to focus on the potential risk of CA-AKI due to drug use, considering the timing that increases CA-AKI risk. In future studies, we need to evaluate this association in more detail, distinguishing the prescription of drugs from related events.

## Methods

### Study subjects and data sources

We used a nationwide cohort with data from the annual health checkup program from 2014 to 2020 obtained from one of the largest employment-based health insurers in Japan. The subjects were civil engineers and employees of construction companies along with their family members aged from 19 to 74 years. We excluded patients with a history of renal failure or eGFR < 15 mL/min/1.73 m^2^ who were on the dialysis or pre-dialysis stage. We also excluded individuals for whom baseline serum creatinine data were unavailable. If creatinine values were missing at the first year that participants were employed, we used the data of the first health checkup along with creatinine values during follow-up as baseline data.

The study complied with the Declaration of Helsinki. The Institutional Review Board of the Kyoto University approved this study (approval number: R0817-2). In accordance with the Japanese Ethical Guidelines for Medical and Health Research Involving Human Subjects, we used anonymised data for analysis^36^. The institutional review board waived the need to obtain informed consent from the participants as we only used anonymised data for analysis in this study. This study followed the Strengthening the Reporting of Observational Studies in Epidemiology (STROBE) recommendations.

### Definition of CA-AKI as the outcome

We defined CA-AKI as the outcome using the ICD-10 codes based on the lists of the London School of Hygiene & Tropical Medicine, London, United Kingdom. We included “Acute kidney failure with lesion of tubular necrosis” (N17.0), “Acute kidney failure with lesion of acute cortical necrosis” (N17.1), “Acute kidney failure with lesion of medullary necrosis” (N17.2), “acute kidney failure with other specified pathological” (N17.8), and “Acute kidney failure, unspecified” (N17.9) (see Supplementary Table S6).

### Extraction of drugs and definition of the prevalent and new drug use

We extracted data on the use of RAAS inhibitors, antihypertensive agents other than RAAS inhibitors, diuretics, antidiabetic agents, PPIs, H2RAs, active vitamin D3 analogues, NSAIDs, and anti-infectious drugs, as drugs prescribed in primary care settings. The drugs were categorised based on the Anatomical Therapeutic Chemical (ATC) classification developed by the World Health Organization (Geneva)^37^. Additionally, we identified drugs without the ATC code using D numbers that distinguish drugs based on their chemical substances (see Supplementary Table S7).

Patients whose prescriptions were within 90 days of a previous prescription of the same drug were treated as prevalent users, while patients whose prescriptions where the previous prescription could not be verified or were prescribed after 90 days even the same drug were treated as new users. The extent to which the drug affected the patient was defined as uniformly within 90 days of the prescription, wherever patients were prevalent users or new users^38^. The patients with contrast medium were not defined as prevalent users because these drugs were not used continuously in the primary care setting. We also did not define NSAIDs and anti-infectious drug users as the prevalent users because they were not continuously prescribed in most cases.

### Other potential risk factors

We extracted baseline data on age, sex, body mass index (BMI), smoking or alcohol habits, regular exercise, blood pressure, past history (stroke, ischaemic heart disease [IHD], chronic heart failure [CHF], and CLD), laboratory data (eGFR, haemoglobin, total protein, uric acid, low-density lipoprotein [LDL]-cholesterol, high-density lipoprotein [HDL]-cholesterol, triglyceride, haemoglobin A1c [HbA1c], and proteinuria) as potential risk factors. eGFR was calculated using the Modification of Diet in Renal Disease study equation modified for Japanese patients: 194 × (serum creatinine)^−1.094^ × (age)^−0.287^ × (0.739 if female)^39,40^. High blood pressure was defined as a systolic blood pressure ≥ 140 mmHg and/or diastolic blood pressure ≥ 90 mmHg based on the corresponding criteria of the Japanese Society of Hypertension 2019 guidelines^41^. Dyslipidaemia was defined as LDL cholesterol ≥ 140 mg/dL, HDL cholesterol < 40 mg/dL, or triglyceride ≥ 150 mg/dL^42^. HbA1c levels were divided into ≥ 6.5% and < 6.5% groups^43^. Proteinuria was evaluated using a dipstick urinalysis, which was categorised as negative, trace, 1 +, 2 +, 3 +, or 4 +. The values of 1 +, 2 +, 3 +, and 4 + were reported to approximately correspond to urine albumin-creatinine ratio values of 30, 100, 300, and 1000 mg/g creatinine, respectively^44–46^. In the present study, positive proteinuria was defined as a proteinuria level ≥ 1 +. We defined several comorbidities including stroke, IHD, CHF, and CLD using the ICD-10 codes based on the lists of the London School of Hygiene & Tropical Drug, London, United Kingdom (see Supplementary Table S6).

### A cohort study for the assessment between the prevalent drug use and CA-AKI incidence

We conducted a cohort study to assess the association between the prevalent drug use and CA-AKI incidence, adjusting for the above-mentioned baseline potential risk factors, including baseline characteristics, comorbidities, and laboratory data (model 1). We identified the prescriptions of RAAS inhibitors, antihypertensive agents other than RAAS inhibitors, diuretics, antidiabetic agents, PPIs, H2RAs, and active vitamin D3 analogues at the baseline as the prevalent drug use.

### A case-crossover study for the assessment between the new drug use and the subsequent CA-AKI incidence

We conducted a case-crossover study to assess the association between the new drug use and CA-AKI incidence^47^. The case period was defined as three months before CA-AKI incidence (− 3 to 0 months), whereas the control period was defined as nine to six months and 15 to 12 months before the CA-AKI incidence in the same participants (see Supplementary Fig. S1). We compared the presence or absence of prescriptions of relevant drugs during the case period to that during the control period. We identified the prescription of contrast medium, NSAIDs and anti-infectious drugs, in addition to that of prevalent drugs included in the cohort study, as the new use of common drugs. We defined the prescriptions of drugs as time-varying covariates, considering that the other potential residual confounders were self-matched by the case-crossover design.

### Statistical analysis

Continuous data on baseline participant characteristics were summarised as means (standard deviation [SD]) for normally distributed variables and medians (interquartile range [IQR]) for skewed variables. Dichotomous and categorical data were described as proportions. We did not summarise baseline data with more than 50% missing values.

First, we estimated hazard ratios (HRs) with 95% confidence intervals (95% CIs) to evaluate the association between the prevalent drug use and CA-AKI incidence using the Cox proportional hazard model. Furthermore, we adjusted for fewer numbers of covariates using sensitivity analyses by considering the limited number of outcomes; the following covariates were selected: age, sex, CKD stage, proteinuria, and drugs for model 2; and age, sex, CKD stage, proteinuria, past histories, and drugs for model 3. Additionally, we estimated cumulative proportions of continued prescription at each time point from year 1 to year 6 using Kaplan–Meier Curves. We calculated the proportion with the denominator: number of drugs at baseline and the numerator: number of drugs continued. Discontinuation of a drug was defined as more than 90 days without a prescription since the last prescription period. We also performed sensitivity analyses, excluding drugs from covariates if the continuation rate of the drugs was < 50% at one year from study initiation and at the median time of follow-up.

Second, we estimated the incidence rate ratios (IRRs) with 95% CIs to evaluate the association between the new drug use and CA-AKI incidence using the conditional Poisson regression model^48^. Furthermore, we compared the use of drugs in the case period with that in the earlier control period (− 15 to − 12 months) as sensitivity analyses, considering the influence of the shortage during the wash-out period.

To handle missing values, multiple imputations using chained equations were performed to impute missing covariates to create 20 copies of data for the cohort study. We subsequently derived the HR with a 95% CI by combining the results from the multiple imputed datasets based on Rubin’s rule^49^. Additionally, we adopted a complete case analysis for the case-crossover study because the missing values regarding drugs were expected to be low.

All statistical analyses were performed using STATA version 16.0 (StataCorp LLC, College Station, TX, USA); a two-sided *p*-value < 0.05 was considered statistically significant.

### Supplementary Information

Supplementary Information.

## Data Availability

The datasets generated and/or analysed during the current study are not publicly available due to the privacy policy of the data provider but are available from the corresponding author on reasonable request.
