# Supplementary material for: Prevalent and new use of common drugs for the incidence of community-acquired acute kidney injury: cohort and case-crossover study
Source: Sci Rep. 2024 Aug 2;14:17906. doi: 10.1038/s41598-024-66532-w (PMC11297046; doi:10.1038/s41598-024-66532-w)
Supplement: Supplementary file 1 — Supplementary Information. [file 41598_2024_66532_MOESM1_ESM.docx]

**Prevalent and new use of common drugs for the incidence of community-acquired acute kidney injury: cohort and case-crossover study**

Miho Kimachi, MD, PhD^1^, Tatsuyoshi Ikenoue, MD, PhD^1,2^, Shingo Fukuma, MD, PhD^1,3, *^

^1^Human Health Sciences, Kyoto University Graduate School of Medicine, Kyoto, Japan

^2^Shiga University Center for Data Science Education and Research, Shiga, Japan

^3^Department of Epidemiology Infectious Disease Control and Prevention, Hiroshima University Graduate school of Biomedical and Health Sciences, Hiroshima, Japan

**Supplementary Table S1. Baseline characteristics of subjects with missing values for creatinine**

| **Characteristics** | **Total**  **(n=78,754)** | **AKI (+)**  **(n=166)** | **AKI (-)**  **(n=78,588)** | **Missing data**  **(n=15,936)** |
| --- | --- | --- | --- | --- |
| Age, year | 53.0 (10.5) | 53.1 (10.0) | 53.0 (10.5) | 56.0 (11.8) |
| Female, n (%) | 20,635 (26.2) | 19 (11.5) | 20,616 (26.2) | 4,704 (29.5) |
| Body mass index | 24.6 (4.0) | 25.1 (5.0) | 24.6 (4.0) | 24.5 (4.1) |
| Smoking, % | 21,733 (27.8) | 72 (43.6) | 21,661 (27.8) | 4,986 (31.8) |
| Alcohol (everyday), % | 26,984 (36.1) | 63 (40.1) | 26,921 (36.1) | 3,933 (35.1) |
| Regular exercise, % | 17,789 (24.3) | 22 (13.9) | 17,767 (24.3) | 2,906 (29.6) |
| Systolic blood pressure, mmHg | 129.7 (18.2) | 132.3 (19.2) | 129.7 (18.2) | 132.5 (19.3) |
| Diastolic blood pressure, mmHg | 80.1 (13.0) | 81.7 (13.2) | 80.1 (13.0) | 80.1 (12.4) |
| Comorbidities, % |  |  |  |  |
| *Stroke* | 2,929 (3.7) | 4 (2.4) | 2,925 (3.7) | 698 (4.4) |
| *Ischaemic heart disease* | 7,108 (9.0) | 16 (9.6) | 7,092 (9.0) | 1,486 (9.3) |
| *Chronic heart failure* | 5,168 (6.6) | 13 (7.8) | 5,155 (6.6) | 1,040 (6.5) |
| *Chronic liver failure* | 15,722 (20.0) | 44 (26.5) | 15,678 (20.0) | 3,356 (21.1) |
| Laboratory data |  |  |  |  |
| *eGFR, ml/min/1.73 m^2^* | 77.4 (15.2) | 76.8 (17.6) | 77.4 (15.2) | - |
| *Creatinine, mg/dL* | 0.80 (0.18) | 0.85 (0.25) | 0.80 (0.18) | - |
| *Haemoglobin, g/dL* | 14.6 (1.5) | 14.5 (1.5) | 14.6 (1.5) | 14.7 (1.5) |
| *Total protein, g/dL* | 7.3 (0.42) | 7.3 (0.51) | 7.3 (0.42) | - |
| *ALT, U/L* | 28.2 (23.0) | 29.6 (22.5) | 28.2 (23.0) | 27.2 (26.7) |
| *AST, U/L* | 25.8 (38.5) | 29.6 (18.1) | 25.8 (38.5) | 26.8 (53.9) |
| *γ-GTP,* | 53.7 (79.7) | 83.9 (157.7) | 53.6 (79.4) | 55.2 (85.6) |
| *Uric acid, mg/dL* | 5.8 (1.5) | 6.0 (1.5) | 5.8 (1.5) | - |
| *LDL cholesterol, mg/dL* | 123.9 (32.1) | 115.6 (38.0) | 123.9 (32.1) | 120.4 (32.6) |
| *HDL cholesterol, mg/dL* | 60.5 (16.9) | 57.6 (17.9) | 60.5 (16.9) | 61.0 (17.1) |
| *Triglyceride, mg/dL* | 132.6 (129.1) | 169.3 (169.2) | 132.5 (129.0) | 139.8 (120.6) |
| *HbA1c, %* | 5.8 (0.88) | 6.0 (1.1) | 5.8 (0.88) | 5.9 (0.93) |
| *urine protein* |  |  |  |  |
| *3+* | 0.39 | 3.0 | 0.39 | 0.74 |
| *2+* | 1.4 | 1.8 | 1.4 | 1.7 |
| *1+* | 4.0 | 7.2 | 4.0 | 4.5 |
| *trace* | 9.1 | 10.2 | 9.1 | 8.0 |
| *negative* | 85.1 | 77.7 | 85.1 | 85.0 |
| prescription, % |  |  |  |  |
| *RAAS inhibitor* | 20,775 (26.4) | 59 (35.5) | 20,716 (26.4) | 5,162 (32.4) |
| *Antihypertensive agent other than RAAS inhibitor* | 24,242 (30.8) | 55 (33.1) | 24,187 (30.8) | 6,058 (38.0) |
| *Diuretic* | 2,923 (3.7) | 7 (4.2) | 2,916 (3.7) | 757 (4.8) |
| *Antidiabetic agent* | 6,248 (7.9) | 22 (13.3) | 6,226 (7.9) | 1,412 (8.9) |
| *Proton pump inhibitor* | 15,113 (19.2) | 32 (19.3) | 15,081 (19.2) | 3,030 (19.0) |
| *Histamine-2 receptor antagonist* | 10,896 (13.8) | 22 (13.3) | 10,874 (13.8) | 2,207 (13.9) |
| *Active vitamin D3 analogue* | 954 (1.2) | 3 (1.8) | 951 (1.2) | 332 (2.1) |

Normally distributed continuous data were summarized as the mean (standard deviation), continuous variables with skewed data were summarized as median (interquartile range) and dichotomous or categorical data were summarized as proportions.

CA-AKI, community-acquired acute kidney injury; eGFR, estimated glomerular filtration rate; ALT, alanine aminotransferase; AST, aspartate aminotransferase; γ-GTP, γ-glutamyl transpeptidase; HbA1c, hemoglobin A1c; and RAAS, renin-angiotensin-aldosterone system.

**Supplementary Table S2. Association between CA-AKI incidence and potential risk factors including the baseline drug use, adjusting for smaller covariates (n=78,753)**

|  | **Model 2** | | | **Model 3** | | |
| --- | --- | --- | --- | --- | --- | --- |
|  | **Hazard ratio** | **95% Confidence interval** | **p-value** | **Hazard ratio** | **95% Confidence interval** | **p-value** |
| Continued use of common drugs |  |  |  |  |  |  |
| *RAAS inhibitor* | 1.64 | (1.07 to 2.52) | 0.024 | 1.60 | (1.04 to 2.46) | 0.032 |
| *Antihypertensive agent other than RAAS inhibitor* | 0.85 | (0.56 to 1.30) | 0.46 | 0.85 | (0.55 to 1.29) | 0.43 |
| *Diuretic* | 0.92 | (0.42 to 2.01) | 0.83 | 0.89 | (0.41 to 1.95) | 0.78 |
| *Antidiabetic agent* | 1.58 | (0.99 to 2.51) | 0.054 | 1.51 | (0.94 to 2.41) | 0.086 |
| *Proton pump inhibitor* | 1.07 | (0.72 to 1.59) | 0.74 | 1.04 | (0.70 to 1.56) | 0.84 |
| *Histamine-2 receptor antagonist* | 1.11 | (0.71 to 1.75) | 0.65 | 1.09 | (0.69 to 1.72) | 0.70 |
| *Active vitamin D3 analogue* | 3.01 | (0.93 to 9.75) | 0.066 | 2.93 | (0.91 to 9.51) | 0.072 |
| Age (every 10 years old) | 1.13 | (0.95 to 1.34) | 0.17 | 1.13 | (0.95 to 1.34) | 0.17 |
| Female | 0.44 | (0.27 to 0.72) | 0.001 | 0.44 | (0.27 to 0.73) | 0.001 |
| eGFR |  |  |  |  |  |  |
| *≥ 90mL/min/1.73 m^2^* | ref |  |  | ref |  |  |
| *60≤ <90 mL/min/1.73 m^2^* | 0.85 | (0.57 to 1.26) | 0.41 | 0.85 | (0.57 to 1.27) | 0.43 |
| *< 60mL/min/1.73 m^2^* | 0.92 | (0.51 to 1.66) | 0.77 | 0.93 | (0.51 to 1.68) | 0.80 |
| Proteinuria | 1.85 | (1.14 to 2.98) | 0.012 | 1.82 | (1.12 to 2.93) | 0.015 |
| Stroke | - | - | - | 0.64 | (0.23 to 1.74) | 0.38 |
| Ischaemic heart disease | - | - | - | 0.98 | (0.56 to 1.71) | 0.95 |
| Chronic heart failure | - | - | - | 1.19 | (0.64 to 2.18) | 0.58 |
| Chronic liver failure | - | - | - | 1.38 | (0.97 to 1.97) | 0.076 |

RAAS, renin-angiotensin-aldosterone system

**Supplementary Table S3. Association between CA-AKI incidence and potential risk factors including the baseline drug use, adjusting for the covariates excluding drugs with a continuation rate <50% one year from beginning the study and at the median time (4.37 years) of the study follow-up (n=78,753)**

|  | **One year from study initiation** | | | **At the median time of study follow-up** | | |
| --- | --- | --- | --- | --- | --- | --- |
|  | **Hazard ratio** | **95% Confidence interval** | **p-value** | **Hazard ratio** | **95% Confidence interval** | **p-value** |
| Continued use of common drugs |  |  |  |  |  |  |
| *RAAS inhibitor* | 1.59 | (1.03 to 2.46) | 0.037 | 1.58 | (1.02 to 2.43) | 0.038 |
| *Antihypertensive agent other than RAAS inhibitor* | 0.87 | (0.57 to 1.34) | 0.53 | 0.89 | (0.58 to 1.35) | 0.58 |
| *Diuretic* | 0.82 | (0.37 to 1.80) | 0.62 | - | - |  |
| *Antidiabetic agent* | 1.37 | (0.80 to 2.34) | 0.25 | - | - |  |
| *Proton pump inhibitor* | 1.04 | (0.69 to 1.56) | 0.85 | - | - |  |
| *Histamine-2 receptor antagonist* | - | - |  | - | - |  |
| *Active vitamin D3 analogue* | 2.96 | (0.92 to 9.58) | 0.07 | - | - |  |
| Age (every 10 years old) | 1.12 | (0.94 to 1.34) | 0.21 | 1.13 | (0.95 to 1.35) | 0.17 |
| Female | 0.26 | (0.14 to 0.48) | 0.001> | 0.27 | (0.15 to 0.49) | 0.001> |
| eGFR |  |  |  |  |  |  |
| *≥ 90mL/min/1.73 m^2^* | ref |  |  | ref |  |  |
| *60≤ <90 mL/min/1.73 m^2^* | 0.97 | (0.65 to 1.46) | 0.89 | 0.97 | (0.64 to 1.45) | 0.87 |
| *< 60mL/min/1.73 m^2^* | 1.02 | (0.55 to 1.89) | 0.95 | 1.03 | (0.55 to 1.90) | 0.94 |
| Proteinuria | 1.70 | (1.04 to 2.78) | 0.033 | 1.71 | (1.05 to 2.80) | 0.031 |
| Stroke | 0.68 | (0.25 to 1.85) | 0.45 | 0.69 | (0.25 to 1.88) | 0.47 |
| Ischaemic heart disease | 1.01 | (0.58 to 1.76) | 0.98 | 1.02 | (0.59 to 1.78) | 0.94 |
| Chronic heart failure | 1.19 | (0.65 to 2.19) | 1.19 | 1.19 | (0.65 to 2.19) | 0.57 |
| Chronic liver failure | 1.40 | (0.98 to 2.00) | 0.067 | 1.43 | (1.01 to 2.04) | 0.046 |
| Body mass index |  |  |  |  |  |  |
| *<18.5* | 3.12 | (1.61 to 6.07) | 0.001 | 3.14 | (1.62 to 6.10) | 0.001 |
| *≥18.5 <25* | ref |  |  | ref |  |  |
| *≥25* | 1.05 | (0.75 to 1.49) | 0.76 | 1.05 | (0.75 to 1.48) | 0.76 |
| Smoking | 1.79 | (1.29 to 2.49) | 0.001 | 1.79 | (1.29 to 2.49) | 0.001 |
| Alcohol (everyday) | 0.94 | (0.68 to 1.31) | 0.73 | 0.94 | (0.67 to 1.30) | 0.70 |
| Regular exercise | 0.55 | (0.35 to 0.87) | 0.01 | 0.55 | (0.35 to 0.88) | 0.012 |
| High blood pressure | 1.06 | (0.76 to 1.48) | 0.72 | 1.06 | (0.76 to 1.47) | 0.75 |
| Haemoglobin | 0.75 | (0.66 to 0.84) | 0.001> | 0.74 | (0.66 to 0.84) | 0.001> |
| Total protein | 1.31 | (0.89 to 1.94) | 0.17 | 1.30 | (0.88 to 1.92) | 0.19 |
| Uric acid | 1.02 | (0.90 to 1.14) | 0.80 | 1.01 | (0.90 to 1.14) | 0.82 |
| Dyslipidemia | 1.17 | (0.85 to 1.62) | 0.34 | 1.17 | (0.85 to 1.62) | 0.34 |
| HbA1c ≥ 6.5% | 1.16 | (0.72 to 1.85) | 0.54 | 1.30 | (0.86 to 1.96) | 0.21 |

CA-AKI, community-acquired acute kidney injury; and RAAS, renin-angiotensin-aldosterone system.

**Supplementary Table S4. Comparison of the new drug use between the case period (-3 to 0 months) and later control period (-15 to -12 months), n=166**

| **Common drugs** | **Incidence rate ratio** | **95% Confidence interval** | **p-value** |
| --- | --- | --- | --- |
| RAAS inhibitor | 1.07 | (0.90 to 1.29) | 0.44 |
| Antihypertensive agent other than RAAS inhibitor | 1.01 | (0.82 to 1.24) | 0.94 |
| Diuretic | 1.32 | (1.10 to 1.58) | 0.003 |
| Antidiabetic agent | 1.01 | (0.81 to 1.24) | 0.96 |
| Proton pump inhibitor | 1.18 | (0.98 to 1.42) | 0.075 |
| Histamine-2 receptor antagonist | 0.99 | (0.78 to 1.25) | 0.92 |
| Active vitamin D3 analogue | 0.86 | (0.60 to 1.22) | 0.39 |
| Non-steroidal anti-inflammatory drug | 1.18 | (0.97 to 1.45) | 0.10 |
| Anti-infectious drug | 1.49 | (1.22 to 1.83) | 0.001> |
| Contrast medium | 1.38 | (1.15 to 1.66) | 0.001 |

RAAS, renin-angiotensin-aldosterone system.

**Supplementary Table S5. Comparison of the relative events and the prescription of drugs between three months immediately before the incidence of CA-AKI and the control periods, n (%)**

| **Events** | **Case** | **Control**  **(Before 6 months)** | **Control**  **(Before 12 months)** |
| --- | --- | --- | --- |
| Stroke | 3 (1.8) | 4 (2.4) | 2 (1.2) |
| Ischaemic heart disease | 11 (6.6) | 1 (0.60) | 0 (0) |
| Chronic heart failure | 18 (10.8) | 1 (0.60) | 1 (0.60) |
| Chronic liver failure | 11 (6.6) | 3 (1.8) | 3 (1.8) |
| Gastroenteritis | 0 (0) | 0 (0) | 0 (0) |
| Gastric bleeding | 15 (9.0) | 1 (0.6) | 1 (0.6) |
| Heatstroke | 15 (9.0) | 0 (0) | 1 (0.6) |
| Sepsis | 16 (9.6) | 0 (0) | 1 (0.6) |

| **Events** | **Case** | **Control**  **(Before 6 months)** | **Control**  **(Before 12 months)** |
| --- | --- | --- | --- |
| RAAS inhibitor | 70 (42.2) | 65 (39.2) | 62 (37.4) |
| Antihypertensive agent other than RAAS inhibitor | 61 (36.8) | 50 (30.1) | 45 (27.1) |
| Diuretic | 27 (16.3) | 5 (3.0) | 6 (3.6) |
| Antidiabetic agent | 27 (16.3) | 20 (12.1) | 17 (10.2) |
| Proton pump inhibitor | 46 (27.7) | 22 (13.3) | 18 (10.8) |
| Histamine-2 receptor antagonist | 18 (10.8) | 11 (6.6) | 12 (7.2) |
| Active vitamin D3 analogue | 3 (1.8) | 3 (1.8) | 3 (1.8) |
| Non-steroidal anti-inflammatory drug | 62 (37.4) | 34 (20.5) | 33 (19.9) |
| Anti-infectious drug | 73 (44.0) | 23 (13.9) | 25 (15.1) |
| Contrast medium | 48 (28.9) | 14 (8.4) | 12 (7.2) |

CA-AKI, community-acquired acute kidney injury.

**Supplementary Table S6. Clinical code list of relevant diseases**

|  | **Clinical code list - ICD-10 for related diseases** | **Citation** |
| --- | --- | --- |
| Acute kidney disease | N17.0-N17.2, N17.8, and N17.9 | Crellin, Mansfield, Leyrat, Nitsch, Douglas, Root, Williamson, Smeeth and Tomlinson (2017). clinical code list - ICD-10 - acute kidney injury. [data collection]. London School of Hygiene & Tropical Medicine, London, United Kingdom. https://doi.org/10.17037/DATA.251. |
| Stroke | I60.0, I61.1, I61.3-I61.6, I61.8-I62.1, I62.9-I63.6, I63.8, I63.9, and I64 | muzambi, R (2020). clinical codelist - Stroke ICD-10 codes. [data Collection]. London School of Hygiene & Tropical Medicine, London, United Kingdom. https://doi.org/10.17037/DATA.00002068. |
| Ischaemic heart disease | I20.0, I20.1, I20.8-I21.4, I21.9-I22.1, I22.8-I23.6, I23.8, I24.0, I24.1, I24.8, I24.9,I25.1, I25.2, I25.5, I25.6, I25.8, I25.9, T82.2, and Z95.5 | Crellin, Mansfield, Leyrat, Nitsch, Douglas, Root, Williamson, Smeeth and Tomlinson (2017). clinical code list - ICD-10 - ischaemic heart disease. [data collection]. London School of Hygiene & Tropical Medicine, London, United Kingdom. https://doi.org/10.17037/DATA.235. |
| Chronic heart failure | I11.0, I26.0, I50.0, I50.1, and I50.9 | Crellin, Mansfield, Leyrat, Nitsch, Douglas, Root, Williamson, Smeeth and Tomlinson (2017). clinical code list - ICD-10 - cardiac failure. [data collection]. London School of Hygiene & Tropical Medicine, London, United Kingdom. https://doi.org/10.17037/DATA.256. |
| Chronic liver disease | B18.1, B18.2, B18.9, K70.0-K70.4, K70.9-K71.3, K71.6-KK72.1, K72.9, K73.0, K73.2, K73.8-K74.1, K74.3-K74.6, K75.0-K75.4, and K75.8-KK76.9 | muzambi, R, Bhaskaran, Smeeth and Warren-Gash (2020). clinical Codelist - Sepsis ICD-10 codes. [data Collection]. London School of Hygiene & Tropical Medicine, London, United Kingdom. https://doi.org/10.17037/DATA.00002062. |
| Enterogastritis | A08.0, A08.3, A08.4, and A09.0 | Forbes (2019). clinical code list - acute gastroenteritis - ICD-10 codes. [data collection]. London School of Hygiene & Tropical Medicine, London, United Kingdom. https://doi.org/10.17037/DATA.00001194 |
| Gastrointestinal hemorrhage | I85.0, K22.6, K25.0-K25.2, K25.4-K25.6, K26.0-K26.2, K26.4-K26.6, K27.0, K28.4, K28.5, K29.0, and K92.0-K92.2 | Iwagami, Tomlinson, Mansfield, Douglas, Smeeth and Nitsch (2018). clinical codelist - ICD-10 GI bleeding. [data Collection]. London School of Hygiene & Tropical Medicine, London, United Kingdom. https://doi.org/10.17037/DATA.00000877 |
| Heatstroke | T67.0-T67.3, and T67.5-T67.8 | Ragettli, Martina; Vicedo-Cabrera, Ana; Flückiger, Benjamin; Röösli and Martin; (2019) Impact of the warm summer 2015 on emergency hospital admissions in Switzerland. environmental health, 18 (1). p. 66. ISSN 1476-069X DOI: [10.1186/s12940-019-0507-1](https://doi.org/10.1186/s12940-019-0507-1) |
| Sepsis | A02.1, A20.7, A22.7, A24.1, A26.7, A32.7, A39.1, A39.2, A40.0-A40.3, A40.8-A41.5, A41.8, A41.9, A42.7, A48.3, B37.7, and R57.8 | muzambi, R, Bhaskaran, Smeeth and Warren-Gash (2020). clinical Codelist - Sepsis ICD-10 codes. [data Collection]. London School of Hygiene & Tropical Medicine, London, United Kingdom. https://doi.org/10.17037/DATA.00002062. |

**Supplementary Table S7. Code list for related drugs**

|  | **Code list - ICD-10 for related drugs** | **General name without ICD-10 codes (D code)** |
| --- | --- | --- |
| RAAS inhibitor | C09 | Alacepril (D01900), olmesartan medoxomil and azelnidipine (D09594), azilsartan and amlodipine besilate (D10524), valsartan and cilnidipine (D10525), telmisartan, and amlodipine and hydrochlorothiazide (D10805) |
| Antihypertensive agent other than RAAS inhibitor | C02A, C02B, C02C, C02D, C02K, C02L, C07AA03, C07AA05, C07AA12, C07AA15, C07AA16, C07AA17, C07AA23, C07AB, C07AG, C07B, C07C, C07D, C07E, C07F, G04CA03, C08C, C10BX03, and C09 | Guanabenz acetate (D00605), azelnidipine (D01145), amosulalol hydrochloride (D01469), aranidipine (D01562), efonidipine hydrochloride (D01604), nipradilol (D01691), arotinolol hydrochloride (D01830), and bunazosin hydrochloride (D01887) |
| Diuretic | C03A, C03B, C03C, C03D, C03E, and C03X | Tripamide (D01895) |
| Antidiabetic agent | A10 | Teneligliptin hydrobromide hydrate (D09756), Tofogliflozin hydrate (D 09978), mitiglinide calcium hydrate Vogliblse (D10160), trelagliptin succinate (D10179), Luseogliflozin hydrate (D10195), Omarigliptin (D10317), Teneligliptin hydrobromide hydrate canagliflozin hydrate (D10897), sitagliptin phosphate hydrate IpragliflozinL-proline (D11064), and anagliptin metformin hydrochloride (D11109) |
| Proton pump inhibitor | A02BC, A02BD |  |
| Histamine-2 receptor antagonist | A02BA |  |
| Active vitamin D3 analogue | A11CC | Falecalcitriol (D01662) and eldecalcitol (D07578) |
| Non-steroidal anti-inflammatory drug | M01AB, M01AC, M01AE, M01AG, M01AH, M01AX01, M02AA, N02BA | Tiaramide hydrochloride (D01341), emorfazone (D01353), ampiroxicam (D01397), zaltoprofen (D01547), loxoprofen sodium hydrate (D01709), mofezolac (D01718), amfenac (D07443), and esflurbiprofen and mentha oil (D10746) |
| Anti-infectious drug | J01, J02, J04, J05, A07A, D01BA, G01AA, P01CX01, and P01BB51 | didanosine (D00296), enviomycin sulfate (D03278), laninamivir octanoate hydrate (D09547), amenamevir (D100654), rabeprazole sodium, amoxicillin hydrate, clarithromycin (D10519), rabeprazole sodium, amoxicillin hydrate, metronidazole (D10520), Vonoprazan fumarate, amoxicillin hydrate, metronidazole (D10774), Vonoprazan fumarate, amoxicillin hydrate, clarithromycin (D10775), and Baloxavir Marboxil (D11021) |
| Contrast medium | V08A |  |

RAAS, Renin-angiotensin-aldosterone system

**Supplementary Figure S1. Time course of the case-crossover design**

**
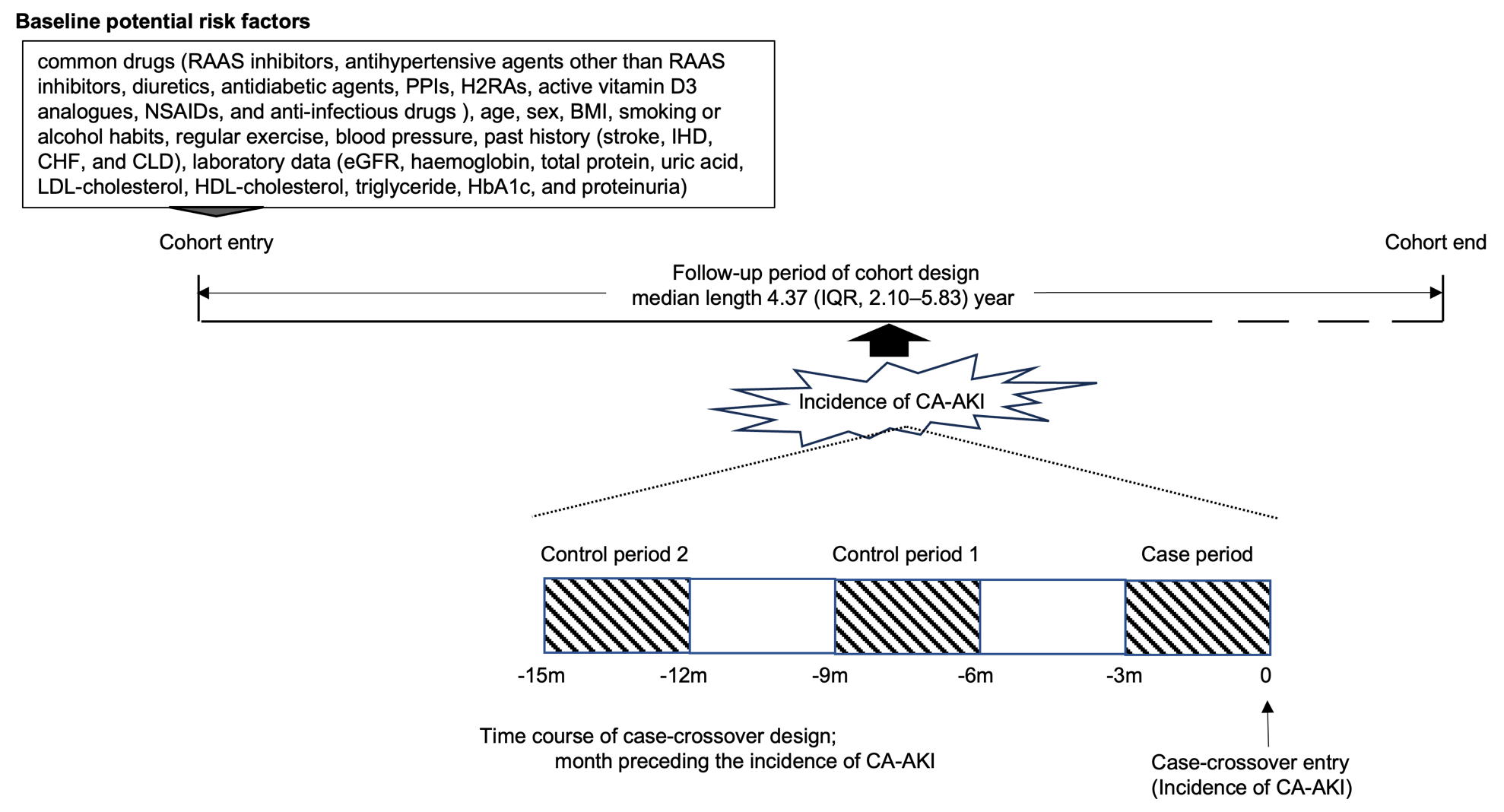
**
